# Recent Advances in the Development of Exogenous dsRNA for the Induction of RNA Interference in Cancer Therapy

**DOI:** 10.3390/molecules26030701

**Published:** 2021-01-29

**Authors:** Tatiana S. Golubeva, Viktoria A. Cherenko, Konstantin E. Orishchenko

**Affiliations:** 1 Department of Genetic Technologies, Novosibirsk State University, Novosibirsk 630090, Russia; CherenkoVA@bionet.nsc.ru (V.A.C.); keor@bionet.nsc.ru (K.E.O.); 2 Federal Research Center Institute of Cytology and Genetics, Siberian Branch of the Russian Academy of Sciences, Novosibirsk 630090, Russia

**Keywords:** RNA interference, exogenous dsRNA, cancer, oligonucleotides, delivery agents

## Abstract

Selective regulation of gene expression by means of RNA interference has revolutionized molecular biology. This approach is not only used in fundamental studies on the roles of particular genes in the functioning of various organisms, but also possesses practical applications. A variety of methods are being developed based on gene silencing using dsRNA—for protecting agricultural plants from various pathogens, controlling insect reproduction, and therapeutic techniques related to the oncological disease treatment. One of the main problems in this research area is the successful delivery of exogenous dsRNA into cells, as this can be greatly affected by the localization or origin of tumor. This overview is dedicated to describing the latest advances in the development of various transport agents for the delivery of dsRNA fragments for gene silencing, with an emphasis on cancer treatment.

## 1. Introduction

**Figure 1:**
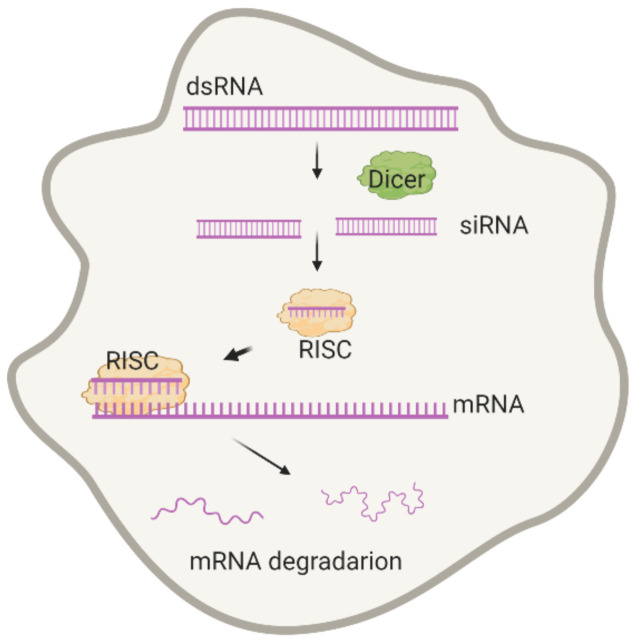
Short scheme of RNAi in cells.

RNA interference is a natural mechanism for gene silencing. It is achieved by cleaving a large double-stranded RNA (dsRNA) precursor into small fragments (21–25 base pairs in length) that act as end effectors through their complementarity to mRNA. The resulting complex is degraded with endonucleases, leading to a reduction in the target mRNA level and a reduction in a synthesis of the corresponding protein. Endogenous siRNAs have not been found in mammals including humans. However, they could be derived from precursor dsRNA (~500 base pairs in length) and small hairpin RNAs (shRNAs) after a cleavage with Dicer or RNAse III (Figure 1). The exploration of this mechanism has made a revolution in the biomedical field. Currently, this approach is used to selectively regulate the activity of specific genes in animals, plants, and humans; as of the beginning of 2020, at least 10 oligonucleotide-based medications have received FDA approval for the treatment of various diseases, including Duchenne muscular dystrophy, spinal muscular atrophy, and cytomegalovirus retinitis [1]. Moreover, gene therapy has other advantages over the conventional treatment. Firstly, it can be administered locally, thereby providing local delivery of a high therapeutic dose without the risk of systemic side effects. Secondly, as most gene therapies are applied on a one-off basis, it can be cost-effective in the long term [2].

RNA interference is applied in many areas of fundamental and practical science including tumor biology. Using a selective knockdown of specific genes (for example, vascular endothelial growth factor (VEGF), c-myc, c-fos), researchers have been able to study the roles of each of them in oncogenesis, and to reveal new factors, which promote or suppress oncogenic cell transformation. The treatment of tumor diseases by RNA interference-mediated therapies also seems to be a very promising approach, as it can be used to selectively knock down almost any gene, thus enabling treatments that account for the patient’s genetic characteristics. Moreover, owing to Watson–Crick pair formation, siRNAs have a significant advantage over the use of monoclonal antibodies or artificially synthesized macromolecules aimed at the recognition of the complex spatial structure of proteins. The latter type of interaction imposes a significant limitation on the use of medications based on antibodies or small molecules, as it is often impossible to identify the target molecule with high activity, affinity, and specificity [1,3]. However, the siRNA method also has its shortcomings: small RNAs degrade quickly under in vivo conditions owing to the abundance of nucleases and phosphatases, a situation that current efforts are endeavoring to solve with chemical modification of oligonucleotides and using various delivery systems based on viral particles, lipids, peptides, exosomes, and inorganic nanoparticles [4,5,6,7,8,9,10,11,12].

This overview describes the main approaches to the delivery of small dsRNA into cancer cells, and discusses the advantages, disadvantages, and prospects of these methodologies in clinical practice (Table 1). Unfortunately, despite a great potential of RNAi application for the therapy of cancer, currently, there are no universal pipelines for a targeted delivery of exogenously synthetized RNA into cells. All of the approaches have multiple limitations such as delivery technique (local vs. systemic), tumor type (solid vs. ascites), and tumor origin (for some tumors, such as hepatocarcinoma, rather effective delivery techniques have been developed).

Currently, a large variety of delivery methods and their modifications have been developed, and there are also a considerable number of target genes to knock down via RNAi. In our review, we attempted to elucidate several common features in already published works within the field of cancer treatment using RNA with a similar object/subject, while it is extremely important in terms of knowledge systematization.

## 2. Viral Particles

### 2.1. Lentiviruses

Lentiviral vectors have the highest transfection efficiency among all viral systems [13]. Being mammalian viruses, lentiviruses can be effectively used to transfer genes into cells [14]. Compared with traditional non-viral delivery vectors, they have many advantages such as high delivery efficiency, speed, and low cost [15]. To date, many viral vectors have been developed for ex vivo and in vivo cell transfection.

In studies on gene silencing in cancer cells, there are many targets for lentiviral, and it is worth mentioning the studies on VEGF (vascular endothelial growth factor) knockdown. Several of them have demonstrated that the inactivation of this gene decreases the rate of cancer cell division in culture, as well as significantly reduces tumor development, angiogenesis, and in vivo invasion in nude mouse xenograft models [16,17,18,19,20].

Diverse microRNA signaling molecules may also be a promising target for inhibition via RNAi by viral delivery systems. MicroRNAs play an important role in the modulation of gene expression as endogenous regulators. Thus, the researchers were able to enhance the apoptosis significantly [21], reduce angiogenesis in tumors [22], diminish the rate of metastasis formation [23], and accelerate differentiation [24].

Also noteworthy is the lentiviral system for efficient overexpression of mutant-template human telomerase RNA [25]. This intervention leads to a significant lengthening of telomeres in cancer cells, which leads to suppression of growth and induction of apoptosis in cancer and precancerous cells. Unfortunately, this method is promising for telomerase-positive cells; no similar effects were observed in telomerase-negative cells.

### 2.2. Adenoviruses

**Figure 2:**
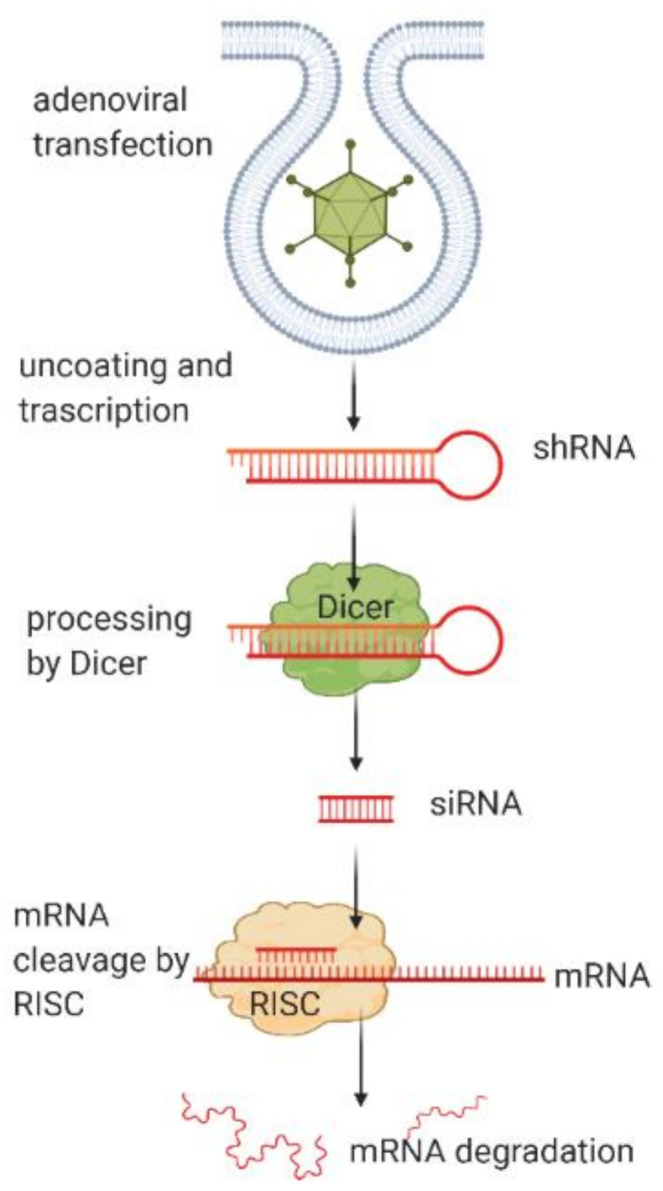
Mechanism of action of adenoviral particle transfection.

Another system for the delivery of target fragments for dsRNA synthesis is the use of adenoviruses (Figure 2), and these have a number of advantages as compared with lentiviruses.

First, the majority of cells in the human body express primary adenovirus receptors and secondary integrin receptors, thus making the adenovirus one of the most effective vehicles for in vivo gene delivery. Secondly, adenoviruses do not integrate their DNA into the host cell genome. Thirdly, despite the safety concerns, adenovirus-based genetic vectors have now been widely used in clinical practice, and currently, safe dosages and injection techniques are already well established. Fourth, adenovirus-based vectors represent a universal platform for the virus capsid modification for the optimization of targeting specificity and other therapeutic characteristics [26].

There has been a concern that their use could be very limited owing to the frequent contact of humans with adenoviruses, resulting in the presence of antibodies that are highly likely to destroy the viral particle before it reaches the target cells. However, there are clinical studies in which oncolytic adenoviruses have avoided destruction by the immune system and successfully reached tumors [27,28]. There are also efforts aimed at the protection of the adenoviral constructs from antibodies and degradation through the use of silicon coating [29]. Such encapsulation of the virus particles significantly improves their distribution and effects in tumor inhibition.

As with lentivirus, search queries reveal a great number of studies covering a wide range of gene targets that were silenced by means of RNA interference. For example, the growth of hepatocellular carcinomas has been significantly inhibited using adenoviral constructs containing fragments able to form short hairpin RNA and miRNA, resulting in a subsequent suppression of the corresponding genes, with the inhibition of cancer cell growth being demonstrated in both cell cultures and in vivo models [30,31,32,33].

There is still a concern about the safety of viruses as vectors for the gene therapy of human diseases. Primarily, the genome regions responsible for the replication of the viral particles are obligatorily deleted from the constructs for safety reasons. However, lentiviruses, for example, typically insert themselves into the genome of the host cell as proviruses, and this can pose a risk of the cell transitioning into a cancerous form. However, these concerns have not been confirmed in any studies to date [34,35]. A hepatotoxic effect has been demonstrated for adenoviruses in the case of intravenous administration, owing to sequestration of the viral particles by the liver, followed by transaminitis and vascular disruption [36,37]. However, no serious adverse effects have been reported in the case of oral adenovirus administration [38]. In general, it should be noted that the local use of viral vectors in gene therapy is preferable to systemic administration, being both a safer and a more effective technique [39].

## 3. Nanoparticles

### 3.1. Lipid-Based Nanoparticles

Lipid-based nanoparticles are used as delivery agents in the first FDA-approved human medication, patisiran (Onpattro**^®^**), which is used to treat amyloid polyneuropathy. Its biologically active substance is a small fragment of modified dsRNA that works via RNA interference. In addition to amyloid polyneuropathy, this delivery method has also been approved for cancer treatment, including combined treatments using dsRNA and chemotherapy [40]. Compared with the viral vectors, this method of siRNA delivery can be used for the systemic administration of medications owing to the high biocompatibility of the lipid-based nanoparticles [41], thus it can be applied for the treatment of both solid and diffuse tissues. This type of nanoparticles usually consists of phospholipids with inlaid by conjugation lipids, which can be modified with various ligands.

It should be noted that lipid-based nanoparticles bear the advantage of being the least toxic for in vivo applications, and significant progress has been made in the area of RNA delivery using lipid-based nanoassemblies. However, this approach has a significant disadvantage of the low tissue selectivity of drug delivery and the low transfection of cancer cells.

First, it is very important to note that glycotargeting, the main method of hepatocyte-targeting, was developed using lipid-based nanoparticles. Targeting unique markers of hepatocytes, asialoglycoprotein receptors, allows to achieve very high results of transfection and inhibition of growth rate in hepatocarcinoma cell lines [42,43]. Moreover, one of the main modifications of nanoparticles is aimed specifically at solving these problems. For example, in hepatocarcinoma therapy, cationic lipid-based nanoparticles can successfully deliver the shNUPR plasmid to suppress the corresponding *NUPR1* gene (which is involved in hepatocellular carcinoma growth and chemoresistance), by protecting the plasmid from DNase I action [40], while building an apolipoprotein crown onto the particle significantly increases its transfection and selectivity to hepatocytes [41]. The same study noted the key roles of both the length of the PEG-conjugated (polyethylene glycol) lipid chain and the amount of PEG in the nanoparticle needed for successful RNA interference therapy of the solid tumors.

Specific modifications in the structure of antibodies have also been used to increase the selectivity of lipid-based nanoparticles for certain types of cells. For example, this approach has worked well with delivering siRNA to lymphocytes, which are normally particularly difficult to transfect with RNA, because they are resistant to traditional transfection reagents and are distributed throughout the body, hindering successful delivery by a systemic administration [44]. In addition to this, a high concentration of reactive oxygen species (ROS) is used as a marker of cancer cells. It facilitates the selective decomposition of the lipid-based carrier to nanoparticles in the cancerous cells [45].

Combined therapy, which includes both a standard cytostatic agent and dsRNA, for silencing a specific gene, within lipid-based nanoparticles, is also a promising approach to the treatment of resistant tumors. An example is the combined delivery of cisplatin together with siRNA, targeting the endonuclease xeroderma pigmentosum group F (XPF), a key of nucleotide excision repair component in mammals. The lipid-based nanoparticles can efficiently encapsulate both cytostatic agents and molecules of siRNA in a specified ratio. Both components are effectively transported into the cells and released therein. As a result, the cisplatin damages the DNA, while the siRNA specifically suppresses the levels of both mRNA and the corresponding XPF protein to enhance the action of the cisplatin, thus leading to increased levels of expression of apoptosis markers and increased cytotoxicity in both cisplatin-sensitive and -resistant cells [46].

### 3.2. Gold Nanoparticles

Gold nanoparticles are also used for stable and safe delivery of various medications, including siRNA. They can be synthesized in a wide range of sizes with diverse surface functionalities. Tunability in size and surface characteristics makes them promising candidates as drug delivery vehicles. Moreover, there is an opportunity for a very precise control over the size, shape, and surface properties of such gold nanoparticles and of their functionalization using various biomolecules [47]. For example, oligopeptides have been used to develop siRNA-delivery systems for the treatment of glioblastomas and breast cancer [48,49]. Unmodified gold nanoparticles possess low transfection efficiency as siRNA delivery agents. However, in vitro experiments have demonstrated a positive role for the incorporation of cations onto the delivery agent surface in order to enhance the uptake of exogenous RNA by cells, for example, functionalizing them with arginine Fe_3_O_4_ [50,51,52,53]. Indeed, for some modifications, the transfection frequency was higher than that seen with the commercial reagent Lipofectamine 2000. Despite this, in vivo experimental results are not so optimistic; that is, systemic administration of cationic delivery systems without biological stabilizing fragments results in their non-specific binding to negatively charged serum proteins, leading to the aggregation and opsonization of the particles. Therefore, PEGylated nanoparticles, having a practically neutral charge, seem more promising for cancer disease therapy [54].

### 3.3. Polymeric Nanoparticles

Polymeric nanoparticles are probably the most diverse category of delivery agents owing to the availability of various polymeric materials. Unlike the abovementioned nanoparticles, the possibilities for their chemical composition and modification are practically unlimited. Moreover, modern materials permit the creation of a 3D nanoparticle structure with the siRNA embedded in it, either throughout the particle or comprising part of its layers. Some materials (especially chitosan-based) are highly promising as oral delivery agents for targeted RNAs. Unlike lipid-based nanoparticles, these delivery agents have no adverse effects on the liver [55].

**Figure 3:**
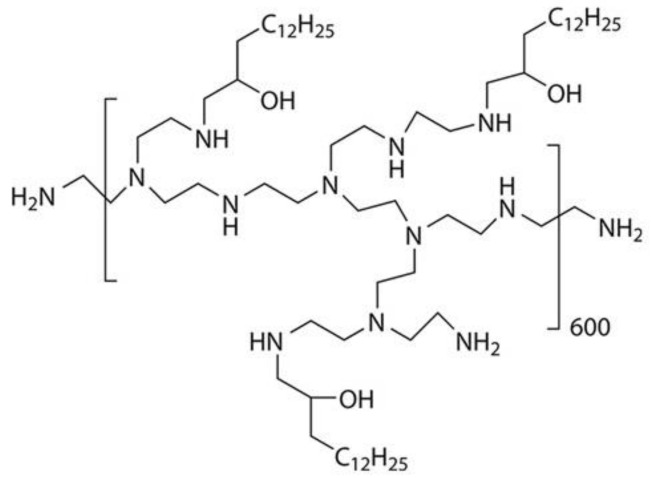
Chemical structure of a 7C1 repeat unit.

Various polymeric materials (gelatin-based, poly(lactic-co-glycolic) (PLGA-modified), cationic amphiphilic) were developed in a series of studies and proved themselves effective as siRNA delivery agents in in vitro systems directed against breast cancer, hepatoma, and myeloid leukemia [56,57,58,59,60]. Despite the fundamentally different nature of the polymer material for the nanoparticles in these studies, they all have the common feature of being positively charged at their surfaces owing to various functional groups aimed at increasing transfection effectiveness. As mentioned above for gold nanoparticles, a positive charge can be a significant disadvantage in the case of systemic administration because of the interaction with serum proteins. In 2018, the first study was published that demonstrated the effect of systemically administered siRNA on nonhuman primates in combination with a 7C1 polymer (Figure 3), low-molecular weight, ionizable polymer that forms nanoparticles [61]. The study provided an extensive histochemical analysis of tissues, showing there were no toxic effects, while the control of cytokines in the blood serum suggested that there were no inflammatory effects in the body. Biochemical blood tests and analysis of the liver function before and after treatment confirmed that the concentrations of proteins and enzymes remained within their normal ranges. The obtained data indicate that the 7C1 complex is a promising siRNA delivery system for systemic administration.

A chitosan-based galactose-modified polymer has also been developed for the oral delivery of siRNA medications [62]. The level of VEGF gene silencing was investigated in mice hepatoma cells, characterized by increased galactose uptake, and thus expected to accumulate an increased concentration of the anti-VEGF siRNA. As a result of the study, significant suppression of the corresponding gene expression, an increase of apoptosis, and an inhibition of angiogenesis have been demonstrated.

Both polymeric nanoparticles and gold nanoparticles are used for combined cancer therapy; for example, when using photocontrolled toxicity to fight cancer cells [63]. In one of the studies, the system of photosensitive polymer nanoparticles included the Pt(IV) prodrug and si (c-fos), thus the release and activation of these components were irradiation-dependent. During exposure to blue light (430 nm), the material was stimulated to release the active components, resulting in cell death. Selective phototherapeutic agents are the basis of an emerging and rapidly developing industry and the use of such medications seems very promising within anti-cancer and anti-bacterial treatment schemes; however, there is a concern related to their photoactivation inside the body because of the low penetrating ability of the radiation used.

### 3.4. Silicon Nanoparticles

Silicon nanoparticles provide an alternative approach to address the maintenance of siRNA integrity while delivering it in the quantities required. The authors previously mentioned the silicon encapsulation of viruses to protect these from the immune system, yet using silicon as an independent delivery agent is much less popular. The main characteristic of silicon that allows it to be considered as a potentially effective siRNA delivery agent is its porosity—in this case, such encapsulation of the RNA molecules protects them from degradation in the body. As with other materials used in nanoparticles, silicon allows for a variety of modifications to enhance uptake selectivity, specifically by cancer cells. For example, it provides for the use of additional peptide or lipid coatings [64]. It has been demonstrated that the amount of siRNA that can be loaded into silicon nanoparticles is significantly affected by the concentration of salts and urea in solution, so this must be taken into account in vivo [65].

## 4. Exosomes and Exosome-Mimetic Nanovesicles

Exosomes (also called “extracellular vesicles”) are natural, nanoscale vesicles that can interact with cell membranes owing to the presence of various adhesive proteins on their surfaces, thus exosomes are considered promising delivery vehicles, also because they are highly biocompatible. This feature provoked many attempts to apply these lipid structures in medicine, and especially in gene therapy for siRNA transport [66]. For example, exosomes were used for silencing of the RAD1 gene, which is one of the main therapeutic targets in cancer treatment [67]. The application of exosomes also enabled to reduce premature ovarian failure, an irreversible effect that women can face after chemotherapy, where anti-apoptotic miRNAs are essential for the restoration of granulosa cells in the follicles. Amniotic fluid can be used as a source of the corresponding exosomes [68].

Taking into account that the cells of multicellular organisms secrete enormous quantities of exosomes, their targeted delivery for gene silencing in cancer tumors is a critical issue. Viral modification of exosomes, as a targeting method, has been approved for RNA delivery in vivo [69]. The main obstacle in the therapeutic application of exosomes is their relatively low yield in any cell culture system and currently complicated purification processes [70].

An alternative to natural exosomes can be artificial exosomes; for example, cell-derived mimetic nanovesicles are a potentially promising alternative to exosomes for clinical applications, demonstrating higher yield without incumbent production and isolation issues [71]. Mimetic nanovesicles could be derived from any cell type. They possess comparable characteristics to exosomes and could be used instead of them. The main source of mimetic nanovesicles is mesenchymal stem cells [72,73]. Firstly, the issues with using mesenchymal stem cells directly are due to their poor engraftment rate, and secondly, there are certain safety problems with their use in humans. Therefore, as an alternative devoid of these shortcomings, mimetic nanovesicles based on exosomes began to be utilized. Artificially synthesized analogs of exosomes have another advantage—their membrane can be modified synthetically in order to obtain optimal physical and chemical properties for purification and release of the contents.

The literature describes the preparation of mimetic nanovesicles using macrophages or macrophages fused with mesenchymal stem cells [72,73,74,75]. Using this technique, it was possible to significantly reduce the proliferation rate of cancer cells in the case of RNAi of the *c-Myc* gene, one of the key regulators of cell proliferation [70,76,77]. In addition to mimetic nanovesicles, the study also used Lipofectamine 2000 and native RNA non-associated with any transport agent as controls. It was shown that the capture of the target RNA occurs equally efficiently with both Lipofectamine 2000 and mimetic nanovesicles, which indicates that they are promising as RNA deliveries for RNAi. It is also worth noting that, when the native RNA was introduced without any delivery system, no RNA interference was detected, suggesting that transport systems are absolutely necessary to protect RNA from degradation.

The complications of the techniques for obtaining nanovesicles are similar to those for exosomes, which are effective delivery vehicles of dsRNA for RNAi. In this regard, it is worth mentioning another study, where researchers proposed to make exosome analogs without the use of cells and to synthesize mimetic nanovesicles completely artificially, in vitro [78]. Using such a methodology, mimetic nanovesicles were obtained based on chitosan nanoparticles covered with a lipid layer that mimics exosomes. Owing to electrostatic interaction, RNA molecules adhered to chitosan particles, while the bilipid layer provided interaction with cells for successful delivery of dsRNA and its protection from degradation. The low toxicity of the developed delivery method is another great advantage, in addition to the possibility of obtaining a large number of mimetic nanovesicles Notably, the toxicity is more than four times lower compared with Lipofectamine 2000. Low transfection efficiency is among the disadvantages of the developed system (lower than lipofectamine). Despite this, the mimetic nanovesicle-mediated delivery system can be very promising for gene therapy thanks to its safety. However, additional research is required for its improvement.

## 5. Peptides

Peptides as siRNA delivery agents may be another promising platform in gene therapy for cancer. They possess flexibility in design, simple compositions and formulations, and diverse physicochemical functions [79]. This delivery system also has drawbacks—peptide agents are very sensitive to proteases, which imposes restrictions on the use of this methodology when peptides are administered systemically. Local administration is preferred for peptide delivery vehicles, however, it is not optimal in the treatment of solid tumors.

First, it is worth noting dendrimers—tree-like polypeptides with a large number of branches. Their branchy structure allows solving several problems at once—part of the molecule is responsible for protecting siRNA from enzymatic degradation, the other part can be functionalized for targeted delivery of RNA molecules to a specific cell type (for example, using antibodies), and additional modifications can be introduced to improve transfection effectiveness. Amphiphilic phospholipid peptide dendrimers successfully delivered siRNA into castration-resistant prostate cancer PC-3 cells [80]. The *Hsp27* gene (heat shock protein 27), one of the main therapeutic targets for the treatment of castration-resistant prostate cancer, was selected to be knocked down. The delivery system had a hydrophobic part based on natural lipids and responsible for interaction with the cell membrane and capture of the vector, and the hydrophilic part consisted of dendritic l-lysin, capable of compacting siRNA into nanoparticles to protect it from enzymatic degradation. In this study, a balance between the hydrophobic and hydrophilic parts of the vector has been achieved, which is reflected in a sufficiently high level of transfection of target cells by the siRNA. In another equally outstanding work, the dendrimer was based on a flexible triethanolamine-core with a polyamidoamine dendritic structure [81]. Here, the researchers applied dual targeting by modifying the dendrimer with additional proteins, interacting with integrin and neuropilin-1 receptors, which led to improved cell penetration, gene silencing, and anticancer activity for the prostate cancer model.

To protect peptides from proteases, an approach using D-isomer amino acids was proposed [82]. The amphipathic peptides created with this methodology demonstrated not only high resistance to proteases, but a capability of self-assembly with siRNA molecules. The researchers note the retention of the basic biophysical characteristics of the retro-inverse form of the protein in comparison with its L-parent homologue. Treatment of cells with the developed complex also produced an effective knockdown of the target gene through RNAi.

Nanocarrier based on aminated poly (α) glutamate was chosen as another promising agent for the systemic use of polypeptides as siRNA delivery technology against solid tumors [83]. The RNA molecules interacted electrostatically with the carrier, leading to the formation of a complex extremely stable in plasma/blood. This approach was approved for systemic administration in vivo against solid tumors—ovarian cancer and lung carcinoma. As a result, a reduction in the expression of the target gene *Rac1* was achieved by 33 and 38%, respectively. The tumor size decreased by 73% and 87%, which indicates the high efficiency and future potential of this approach for the treatment of solid tumors.

## 6. Conjugates

Among the conjugates for targeted delivery of oligonucleotides, givosiran is currently the standard. It is the second drug after patisiran approved by the FDA for the treatment of acute hepatic porphyria [84]. Givosiran is a small interfering RNA (siRNA) directed toward the 5-aminolevulinic acid synthase, an important enzyme in the production of heme part in hemoglobin. It is covalently bound to a ligand containing three *N*-acetylgalactosamine residues that facilitate uptake into hepatocytes via asialoglycoprotein receptors, which are highly expressed on the cell surface of hepatocytes and are selective for glycoproteins containing *N*-acetylgalactosamine residues [85]. It is worth highlighting *N*-acetylgalactosamine as a very promising molecule for the delivery of siRNA to hepatocytes; in addition to the already approved givosiran, there are another seven conjugates in registrational review or phase 3 trials and at least another 21 conjugates at earlier stages of clinical development [86]. Additional enhancements, such as hexopyranose chemical modification altriol nucleic acid within siRNA, significantly enhanced the protection of the oligonucleotide against 5**’**-exonuclease degradation [87]. Studies on the targeted delivery of siRNA through modifications using *N*-acetylgalactosamine residues are sufficient for a separate full review; in this review, only a small part of them is considered.

An example of a successful approach can be the conjugation of siRNA with docosanoic acid to target myostatin, a key determinant of muscle loss and cachexia in cancer, in skeletal and cardiac muscle cells [88,89]. The siRNAs delivered in this way provided more than 55% of gene silencing in muscle cells and about 80% in cardiac cells, increasing tissue volume by more than half.

Conjugation of siRNA with cholesterol allows to neutralize the negative charge of RNA and, consequentially, the impermeability of the cell membrane for it. In one of the studies, 356 cholesterol-conjugated siRNA molecules with various additional modifications were tested, and an algorithm that effectively predicts the activity of miRNA was developed based on linear regression approach [90]. As a result, conjugates were obtained that effectively transfect cancer cells, the chemical modifications of which were predicted by the algorithm. This approach eliminates the need for stochastic screening and optimizes the development of delivery systems.

## 7. Conclusions

This overview is an attempt to summarize the main trends in siRNA delivery in the field of cancer disease therapy. Taking into account the rapid development of gene therapy in general, and the possibilities for individual gene regulation using RNA interference, the authors identified a great number of studies from which it was extremely difficult to draw general conclusions to produce a coherent overview.

Summarizing the work performed in this field, the authors can declare that RNA interference has a promising future in cancer treatment, and that the number of approaches to the targeted delivery of siRNA will only increase. The limitations of the existing approaches will eventually be overcome, enabling the selection of optimal delivery systems for exceptionally effective gene therapy of oncological diseases.

## Figures and Tables

**Table 1:** Short summary of methods for exogenous RNA delivery.

Type of Delivery	Advantages	Disadvantages
1. Viral particles		
- lentiviruses	High delivery efficiency, speed, and low cost	DNA integrating into the host cell genome
- adenoviruses	Adenoviruses do not integrate their DNA into the host cell genome	Low transfection efficiency, the presence of antibodies that are highly likely to destroy the viral particle before it reaches the target cells
2. Nanoparticles		
- lipid-based nanoparticles	Can be used for the systemic administration of medications owing to the high biocompatibility, can be applied for the treatment of both solid and diffuse tissues	Low tissue selectivity of drug delivery and the low transfection of cancer cells
- gold nanoparticles	Very precise control over the size, shape and surface properties	Low transfection efficiency as siRNA delivery agents
- polymeric nanoparticles	Possibilities for their chemical composition and modification are practically unlimited	Low tissue selectivity of drug delivery and relatively low transfection of cancer cells
- silicon nanoparticles	Silicon encapsulation of dsRNA protects them from the degradation	The amount of siRNA that can be loaded into silicon nanoparticles is significantly affected by the concentration of salts and urea
3. Exosomes and exosome-mimetic nanovesicles	High biocompatibility	Relatively low yield in any cell culture system and currently complicated purification processes
4. Peptides	Flexibility in design, simple compositions and formulations, diverse physicochemical functions	Peptide agents are very sensitive to proteases, which imposes restrictions on the use of this methodology when peptides are administered systemically
5. Conjugates	High biocompatibility and low toxicity	Low tissue selectivity of drug delivery and the low transfection of cancer cells

## Sample Availability

Not available.

## Abbreviations

dsRNA	double-stranded RNA
siRNA	small interfering RNA
RNAi	RNA interference
shRNA	short hairpin RNA
